# Photometric-Stereo-Based Defect Detection System for Metal Parts

**DOI:** 10.3390/s22218374

**Published:** 2022-11-01

**Authors:** Yanlong Cao, Binjie Ding, Jingxi Chen, Wenyuan Liu, Pengning Guo, Liuyi Huang, Jiangxin Yang

**Affiliations:** 1 State Key Laboratory of Fluid Power and Mechatronic Systems, School of Mechanical Engineering, Zhejiang University, Hangzhou 310058, China; 2 Key Laboratory of Advanced Manufacturing Technology of Zhejiang Province, School of Mechanical Engineering, Zhejiang University, Hangzhou 310058, China; 3 Zhejiang Academy of Special Equipment Science, Zhejiang University, Hangzhou 310023, China; 4 Key Laboratory of Special Equipment Safety Testing Technology of Zhejiang Province, Hangzhou 310023, China

**Keywords:** photometric stereo, defect detection, convolutional neural network

## Abstract

Automated inspection technology based on computer vision is now widely used in the manufacturing industry with high speed and accuracy. However, metal parts always appear in high gloss or shadow on the surface, resulting in the overexposure of the captured images. It is necessary to adjust the light direction and view to keep defects out of overexposure and shadow areas. However, it is too tedious to adjust the position of the light direction and view the variety of parts’ geometries. To address this problem, we design a **p**hotometric-**s**tereo-**b**ased **d**efect **d**etection **s**ystem (PSBDDS), which combines the photometric stereo with defect detection to eliminate the interference of highlights and shadows. Based on the PSBDDS, we introduce a photometric-stereo-based defect detection framework, which takes images captured in multiple directional lights as input and obtains the normal map through the photometric stereo model. Then, the detection model uses the normal map as input to locate and classify defects. Existing learning-based photometric stereo methods and defect detection methods have achieved good performance in their respective fields. However, photometric stereo datasets and defect detection datasets are not sufficient for training and testing photometric-stereo-based defect detection methods, thus we create a **p**hotometric **s**tereo **d**efect **d**etection (PSDD) dataset using our PSBDDS to eliminate gaps between learning-based photometric stereo and defect detection methods. Furthermore, experimental results prove the effectiveness of the proposed PSBBD and PSDD dataset.

## 1. Introduction

**Figure 1:**
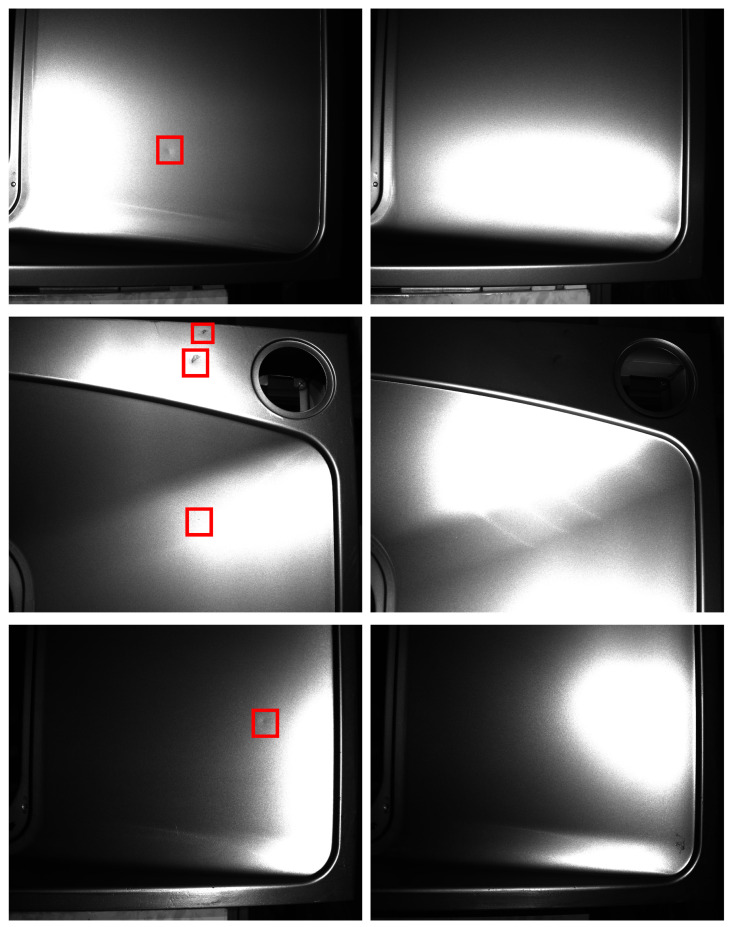
Images of stationary metal objects under different lighting conditions. Each row represents images of an object captured under different directional light. The red box indicates the location of the defect. (**Left**): defect visible. (**Right**): defect in overexposed or shadow area.

Quality control is an essential part of the manufacturing industry, which helps standardize both production and reactions to quality issues. Most of the current inspection work is mainly done manually by visual inspection. The results are heavily influenced by subjective human factors, leading to unstable production quality control. The development of computer vision has greatly alleviated this problem. Automated inspection technology based on computer vision is one of the popular ways for efficient and high-precision defect detection through processing and analysing the images. Traditional defect detection methods based on computer vision mainly use techniques such as edge detection [1,2], grey-scale thresholding [3,4], and image segmentation [5,6]. However, traditional ones have limitations such as their reliance on artificially defined features. In recent years, numerous learning-based techniques have been proposed for defect detection, which transform data into complex and abstract representations that enable the features to be learned during the training phase to overcome the requirement of predefined features. A necessary condition for defect detection using computer vision algorithms is the ability to extract the characteristics of the defects in the image. However, the high reflective properties of the metal surfaces cause overexposure in some areas of the captured image and override the characteristics of the defect, as shown in Figure 1.

When the angular bisector of the incident light and the view coincide with the normal vector, the observation points appear glossy. The surface prevents the light from falling on itself, which leads to the attached shadow in the observation area. When the surface prevents the light from falling on another surface, the observation are appears with a cast shadow [7]. However, as the shape of the object is unknown, the way to avoid defects being in the highlight or shadow areas is to adjust the angle between the direction of light incidence and the direction of view. However, it is too tedious to adjust the light direction and the view with the variety of shapes of metal parts. It is therefore effective and convenient to reduce the probability of defects remaining in the highlight or shadow areas by illuminating the object in different directions. Furthermore, we introduce photometric stereo in the metal defect detection task, which removes the interference of highlights by using the information from multiple illuminations.

In recent years, photometric stereo has received significant attention in the field of advanced manufacturing [8,9,10,11,12], which estimates the accurate and highly detailed surface normal of a target object based on a set of images captured in different light directions using a fixed-viewpoint camera. The basic theory of photometric stereo was first proposed by Woodham et al. [13] based on the assumption of ideal Lambertian reflectance in the 1980s. However, there are few objects in reality that fit the ideal Lambertian assumption. Therefore, over the last forty years, numerous methods aimed at non-Lambertian objects have been proposed to improve the applicability of photometric stereo. Before the development of deep learning, most traditional photometric stereo methods treated the non-Lambertian pixels as outliers [14,15,16,17,18]. However, these methods only worked for objects with sparse non-Lambertian regions. To handle dense non-Lambertian reflections, some methods used analytical or empirical models to simulate reflection on the surface models [19,20,21,22,23,24,25]. However, this type of method is only applicable to a limited range of materials. In recent years, numerous deep-learning-based photometric stereo methods have been proposed to handle non-Lambertian objects in reality. According to how learning-based methods process the input image, methods can be divided into per-pixel [26,27,28,29] and all-pixel methods [30,31,32,33]. Per-pixel methods take the observed intensities as the input and output a surface normal for a single pixel. Santo et al. [34] first introduced deep learning into photometric stereo and propose a deep photometric stereo network (DPSN), which assumed that the light direction stayed the same during the testing and training. To generalize the learning-based per-pixel methods, Ikehata projected the observation intensities onto a 2D space and proposed an observation map, which rearranged the observation intensities according to light directions. The operation of the observation map enabled per-pixel methods to handle the arbitrary number of directions and order-agnostic lighting. Numerous methods, such as CNN-PS [26], LMPS [28], and SPLINET-Net [29], used this data preprocessing method in their neural networks and achieved great performance on the DiLiGenT benchmark dataset. All-pixel methods take whole images or patches as well as their corresponding light directions as input and output a surface map with the same resolution as the input. To handle order-agnostic light, Chen et al. [30] proposed an all-pixel photometric stereo network named PS-FCN, which used a sharing-weight feature extractor to handle per-input image and used max pooling to aggregate the extracted features from multiple images. Based on this strategy, SDPS-Net [32] and GC-Net [35] were proposed to handle the uncalibrated photometric stereo task. According to the summary by Zheng et al. [36], per-pixel methods are robust to nonuniform distributions of surface materials but cannot handle well regions with global illuminations effects (shadows or inter-reflection) since shape information is not explicitly considered. Because all-pixels methods are generally trained using various shapes with a uniform material for each shape, they perform well for regions with a global illumination effect, while they are ineffective for objects with nonuniform materials. There are several methods to improve performance by combining the per-pixel and all-pixel methods. SPS-Net used the attention mechanism to extract photometric information, then applied convolution layers to extract spatial information. Yao et al. [37] proposed a graph-based photometric stereo network (GPS-Net) that first introduced structure-aware graph convolution filters to explore per-pixel information and classical convolution to explore spatial information. Ikehata [38] applied Transformer [39] to extract per-pixel and spatial information and proposed PS-Transformer, which aimed to handle the sparse photometric stereo task. Both defect detection methods and photometric stereo methods achieve outstanding performance on their respective benchmark. There are also methods [8,9,40,41] that combine photometric stereo with defect detection. Ren et al. [42] used a data-driven PS method to extract the surface normal and separate defects from the background through filters. The method could only locate defects but not classify them and could not be applied when the defects and the background had relatively similar characteristics in the frequency domain. Feyza et al. [43] used an L2 photometric stereo [13] method to estimate the albedo map and normal map of objects. Furthermore, they took the combination of the albedo map and the normal map as input and used a CNN to detect the defects. The L2 photometric method is based on the Lambertian assumption and does not apply to most objects in reality. Defect detection and photometric stereo methods have achieved outstanding performance on their respective benchmarks with the development of deep learning. However, there are few methods that combine learning-based photometric stereo methods with learning-based defect detection methods. This is because most of the current defect detection data are captured under a single illumination and cannot be applied to photometric stereo. Meanwhile, existing photometric stereo datasets lack defect samples.

To address this problem, we design a **p**hotometric-**s**tereo-**b**ased **d**efect **d**etection **s**ystem (PSBDDS) to detect defects using photometric information under multilight illumination and create a **p**hotometric **s**tereo **d**efect **d**etection dataset (PSDD) for combining existing defect detection networks with photometric stereo networks. Compared to existing methods, our method can estimate the most realistic objects with non-Lambertian surfaces while being effective in locating and classifying multiple types of defects. We first estimate the normal map from images, captured by the PSBDDS, through the non-Lambertian photometric stereo network and feed the normal map into the defect detection network. In summary, the contributions of this paper are as follows. (1) We build a photometric stereo defect detection dataset for the integration of existing photometric stereo methods with defect detection methods. (2) We design a photometric-stereo-based defect detection system to collect photometric data on the surface of metal parts and apply the photometric-stereo-based defect detection method. (3) We experimentally validate that our dataset can be used to combine the state-of-the-art photometric stereo methods with the advanced defect detection methods for highly accurate and robust defect detection performance.

## 2. Method

### 2.1. Photometric-Stereo-Based Defect Detection System (PSBDDS)

PSBDDS consists of hardware and control modules.

**Figure 2:**
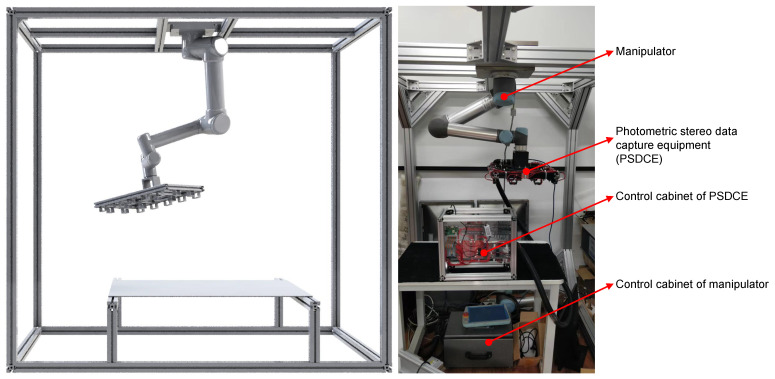
The diagram of our PSBDDS. The image on the left is a 3D model and the image on the right is a real device. The manipulator is mounted on a frame made of aluminium profiles and the PSDCE is attached to the end of the manipulator for capturing photometric stereo. The PSDCE and the manipulator are controlled by their corresponding control cabinets.

**Figure 3:**
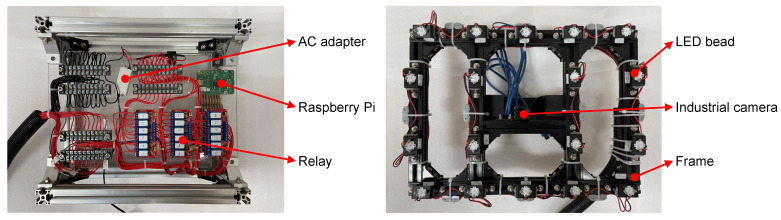
Diagram of the PSDCE and its control cabinet. The image on the left is a control cabinet. We used the Raspberry Pi to control the LED. As the IO port voltage of the Raspberry Pi cannot meet the needs of all LED beads, we used an AC adapter in conjunction with a relay to power LED. The image on the right is the PSDCE. The frame has been blackened to prevent reflective interference. The LED beads are mounted on the frame and the industrial camera is mounted at the centre of the frame.

**Hardware.** Our hardware module consisted of photometric stereo data capture equipment, a manipulator, and a profile frame, as shown in Figure 2. We mounted the PS data capture equipment at the end of a six-degree-of-freedom manipulator, which could adjust the posture and trajectory to capture data at target points. As shown in Figure 3, the photometric stereo data acquisition device consisted of 18 LED beads and an industrial camera. The inspection system of photometric stereo needs to take several pictures in a short time. We thus chose industrial cameras equipped with CMOS sensors with their advantages in high-speed imaging. We set the inspection area size to $v_{x}=150$ mm, $v_{y}=120$ mm and the inspection accuracy to 0.5 mm. Based on our experience in the design of computer-vision-based defect detection systems, 5∼10 pixels were required to characterize the accuracy of the detection. Therefore, the corresponding pixel accuracy was approximately 0.05∼0.1 mm. We took the middle value of the pixel accuracy $a_{pixel}=0.08$ mm for calculating the resolution of the camera.

(1) $$\begin{array}{c} p_{x}=\frac{v_{x}}{a_{pixel}}=\frac{150}{0.08}=1875 \end{array}$$

(2) $$\begin{array}{c} p_{y}=\frac{v_{y}}{a_{pixel}}=\frac{120}{0.08}=1500 \end{array}$$

where $p_{x}$ and $p_{y}$ represent the minimum length and width of the resolution. According to the calculation result, we chose an industrial camera of 5 megapixels and a 2448×2048 resolution. We chose a fixed-focus lens with a focal length of 25 mm and an aperture range of f2.8∼16. The light source was also a key part of the data acquisition system. Following the general settings on the photometric stereo, we used the power of 3 W LED beads as the light source and installed them evenly in a rectangle frame; the aspect ratio of the frame was the same as the camera’s field of view.

**Figure 4:**
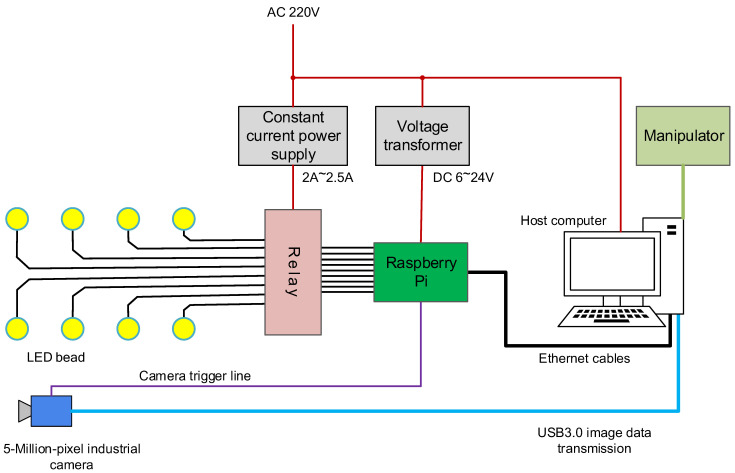
The control scheme for hardware. The host computer controls the communication among the manipulator, the Raspberry Pi, and the industrial camera. The Raspberry Pi controls the operation of the LED beads.

**Control modules.** The control system of the defect detection system was divided into two parts: the control system of the PSDCE, as shown in Figure 3 and the control of the manipulator. The manipulator had existing control boxes. After establishing a connection with the host computer, they received manipulator control commands sent from the host computer and controlled the manipulator to achieve fast and accurate movement. Figure 4 shows the control scheme for hardware. We chose to use a Raspberry Pi as the controller between the PC and the photometric stereo data capture equipment, using the GPIO pins to control the switch of the LEDs and send the data capture command to the camera. The industrial camera had an external trigger line that could be connected directly to the GPIO pins of the Raspberry Pi for image capture triggering. The Raspberry Pi used an Ethernet interface for data interaction with the host PC. A TCP/IP connection between the host computer and the device was established via the socket interface to enable information transfer. However, the output voltage of the GPIO pins of the Raspberry Pi could not meet the demand for LED power. We thus used a constant current supply to provide a steady current and GPIO control relays to switch the circuit.

### 2.2. Dataset

**Figure 5:**
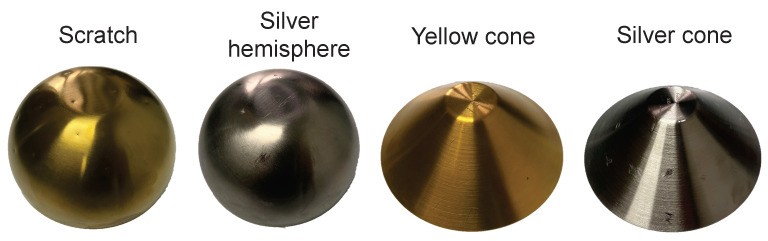
Objects for datasets. The dataset contained two colours (yellow and silver) and two shapes (hemisphere and cone) of objects. The material of the objects was 304 stainless steel. The two objects on the left are hemispheres with a diameter of 16 cm and a height of 5 cm. The two on the right are cones with a diameter of 15 cm and a height of 6 cm.

We used four types of objects to produce our dataset, namely *yellow hemisphere*, *silver hemisphere*, *yellow cone*, and *silver cone*, as shown in Figure 5. The objects contained both curved and flat samples, made of 304 stainless steel and had a wide range of high-gloss areas on the surface. We created defects on the surfaces of objects by simulating the impact of the production process. We used 18 different directional lights to illuminate the object and captured the observed images with a resolution of 400×400. The photometric stereo model used images or pixels under different illuminations as input to estimate the normal map, thus processing 18 high-resolution images together would consume huge computational resources as well as significantly slow down the computation. Most of the existing defect detection methods generate region proposals through enumeration or sliding windows or split the image into patches for processing, thus the high-resolution images would also consume a lot of time for the detection method.

**Figure 6:**
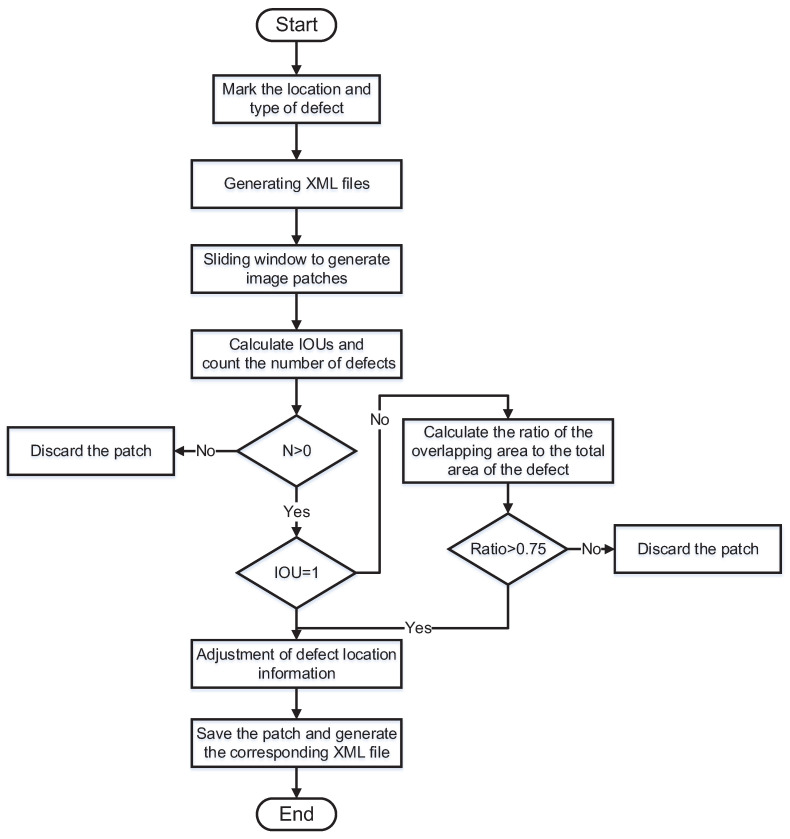
Flowchart of dataset processing. First, we use the labelling tool LabelImg to label the rectangular box in the target area of the defect. We experimentally set the initial size of the sliding window to 400×400 and the step size to 200. The algorithm reads the annotation file of each image, gets the location and category information for each defect in the image, and traverses the image starting from the top left corner of the image. The algorithm first determines whether there is a defective target in the area of the window. If there is no defect, the cropped image is discarded. Otherwise, the defect area information is saved. Then, the algorithm determines whether there is a window containing an incomplete defect. If not, the defect location information is converted to the image coordinate system of the window and saved to generate a new annotation file. When there is an incomplete defect area, the algorithm calculates the ratio of that defect area in the window to the area of the actually marked defect. When the ratio is greater than 0.75, the annotation information of the defect is adjusted and the window boundary is used as the new boundary. When the ratio is less than 0.75, the defect is discarded, and the window image is then saved and a new annotation file is generated.

**Figure 7:**
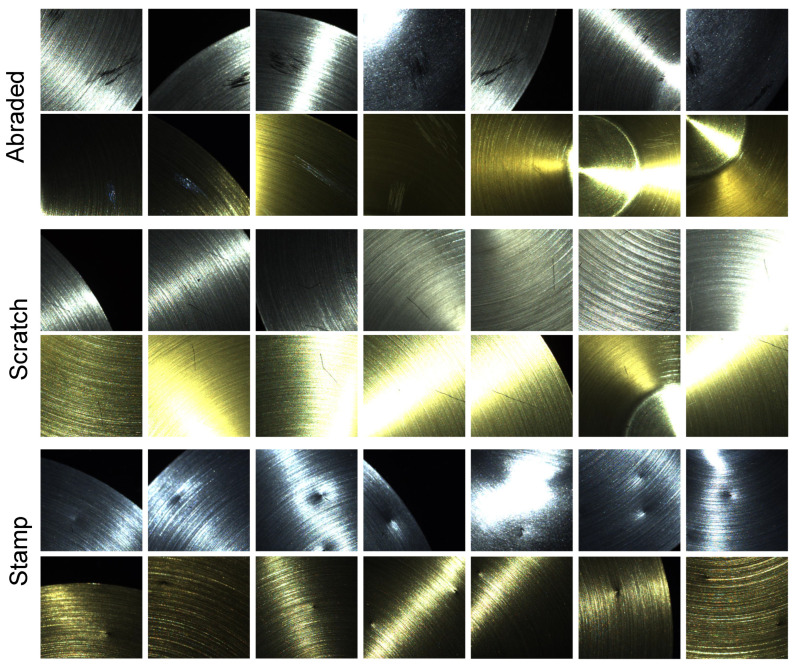
Diagram of the defects. Our dataset contains three types of defects. Rows 1 and 2 are samples of abraded defects. The abraded defects occur in patches and are irregular in shape. Rows 3 and 4 are samples of scratch. The scratches appear individually and are narrow and long. Rows 5 and 6 are samples of the stamp. Stamps usually appear to be circular, with a significant variation in depth.

To improve the efficiency of the detection, we used sliding windows to split the whole image into multiple low-resolution images. The process of making the dataset is shown in Figure 6. The split images are shown in Figure 7. We obtained a total of 2017 defective samples, including 924 **stamp** samples, 542 **scratch** samples, and 551 **abraded** samples. The minimum **stamp** size was 0.45×0.87 mm, the minimum **scratch** size was 0.39×2.21 mm, and the minimum **abraded** size was 1.73×2.78 mm. Each sample contained 18 images with a corresponding light direction and intensity. We divide the dataset into a training set, a validation set, and a test set based on a 3:1:1 split. The number of samples in each category is shown in Table 1.

### 2.3. Detection Framework

**Figure 8:**
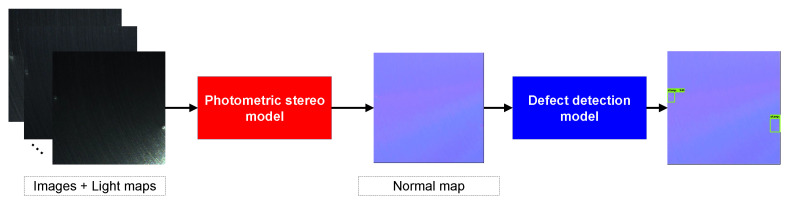
The framework of our detection model. The images captured under different illumination and the corresponding light information are used as input and the normal map is estimated through the photometric stereo model. The defect detection locates and classifies defects with the input of the normal map.

As illustrated in Figure 8, our detection framework consisted of two components, the photometric stereo module and the defect detection module. The photometric stereo module took captured images and corresponding light maps as input and estimated the normal map. Then, the defect detection module used the normal map as input to locate and classify defects. We chose MT-PS-CNN [44] as our photometric stereo model for estimating normal maps. Compared to all-pixel methods (i.e., PS-FCN [30], SDPS-Net [32]) and per-pixel methods (i.e., DPSN [34], CNN-PS [26]), MT-PS-CNN combines the advantages of both types of methods through a 3-D convolution, effectively eliminating the interference highlights, and it performs well with sparse inputs. For a detailed description of MT-PS-CNN, please refer to [44].

**Figure 9:**
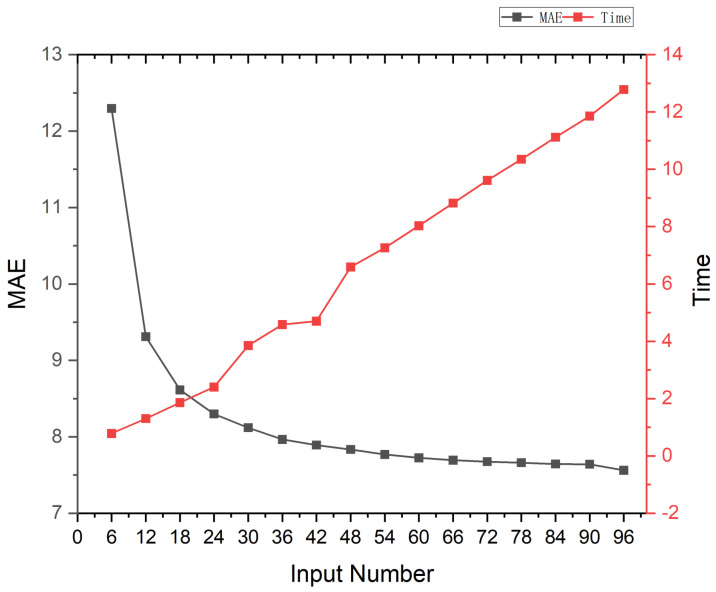
Performance and prediction time of MT-PS-CNN with the different number of inputs on the DiLiGenT dataset. The horizontal axis represents the number of inputs. The left vertical axis represents the mean angular error (MAE °). The right vertical axis represents the prediction time (s). Beginning with 96 inputs, we reduced the number of inputs by 6 at a time and calculated the average MAE over 10 trials.

The number of LED beads has a huge impact on the estimation of the normal map. More LED beads mean more valid clues. However, a large number of image inputs reduces the efficiency of the model computation. Therefore, we analysed the performance of MT-PS-CNN with different numbers of inputs to find a trade-off between the accuracy of the normal estimation and computational speed, as shown in Figure 9. We found that the normal estimation error rose rapidly when the number of inputs was less than 18. The model prediction time grew linearly with the number of inputs. Combining photography time, computational resource consumption, normal solution accuracy, and model prediction speed, we finally chose 18 images as our inputs and set up 18 LED beads.

Existing defect detection methods are mainly divided into one-stage (i.e., YOLO [45,46,47], SSD [48], RON [49]) and two-stage methods (i.e., SPP-Net [50], Fast R-CNN [51], Faster R-CNN [52]). The two-stage approach divides the detection problem into two stages, first generating region proposals, then adjusting the position of the generated region and using the information within the region for classification. The two-stage method is highly accurate but slower than one-stage methods. One-stage methods directly predict the bounding boxes and object classification through a single network. Compared to two-stage methods, one-stage methods are faster but less accurate. In our detection task, the method needs to detect tiny defects in a large background area, and the requirement for detection accuracy is higher than the speed.

The detection speed is similar for both types of methods running on a high-performance computer. For balance, we chose the representative and effective two-stage method, Faster R-CNN, as the basis detection network. The Faster R-CNN model is divided into four parts: the feature extraction module, the region proposal network (RPN), the ROI pooling layer, and the target prediction module. Taking the normal map as input, the Faster R-CNN acquires the feature map using a feature extractor. The RPN generates the region proposals: generating anchor boxes with the pixels of the feature map and selecting and correcting the position of anchor boxes to obtain region proposals. The ROI pooling layer acquires the feature maps according to the region proposals and resizes the features maps into fixed-size feature vectors through the pooling operation. The target prediction module performs class classification and location regression based on feature vectors. For a detailed description of Faster R-CNN, please refer to [52].

## 3. Results

All experiments were performed on a GeForce RTX 2080Ti and 64GB RAM. The SGD optimizer was used to optimize our network with a learning rate of $2.5\times 10^{-3}$, momentum of 0.9, and weight decay of $1\times 10^{-4}$. The training process took 20 epochs with a batch size of 32.

### 3.1. Performance Criteria

We used the Intersection over Union (IoU) to evaluate the overlap between the ground-truth bounding box and the predicted bounding box, which can be expressed as

(3) $$\begin{array}{c} \mathrm{IoU}=\frac{P\cap G}{P\cup G}, \end{array}$$

where P is the predicted bounding box and G represents the ground-truth bounding box. The range of IoU is from zero to one where zero means that there is no overlap between the predicted and ground-truth regions and one means both regions overlap perfectly. According to common practice [53,54,55], we set the IoU threshold to 0.5. The sample with a threshold greater than 0.5 was positive, while the opposite was a negative sample. We applied the mean average precision (mAP) to evaluate the detection accuracy of the model. The confusion matrix was used to summarize the classification results; taking binary classification as an example, the target sample was positive, and the nontarget sample was negative, as shown in Table 2.

The ratio of positive samples correctly predicted to total samples is called the precision.

(4) $$\begin{array}{c} \mathrm{Precision}=\frac{TP}{TP+FP} \end{array}$$

The ratio of positive samples correctly predicted to total positive samples is called the recall.

(5) $$\begin{array}{c} \mathrm{Recall}=\frac{TP}{TP+FN} \end{array}$$

The mAP is the mean of the average precision (AP) for each category. The mAP for the *N* categories can be expressed as:

(6) $$\begin{array}{c} mAP=\frac{1}{N}\sum_{i=1}^{N}AP_{i} \end{array}$$

where AP indicates the average precision of one category, which is the area under the precision–recall curve.

### 3.2. Detection Performance Analysis

The backbone of Fast R-CNN was used to extract features from input images. The performance of Fast R-CNN with different backbones varied considerably. We compared the performance of three mainstream backbones: VGG-16 [56], ResNet-50 [57], ResNet-50+FPN [58], as shown in Table 3.

The introduction of the FPN (feature pyramid network) structure in the backbone can effectively retain shallow detail information and improve performance. Thus, we chose ResNet-50+FPN as the backbone of Fast R-CNN. Taking the normal map as input, we also trained representative one-stage defect detection methods (SSD [48] and a YOLO-V3 [55]) network. Table 4 shows the performance of one-stage and two-stage approaches on the test dataset.

Faster R-CNN achieved good performance through two-stage operations and FPN that made full use of shallow local information and deep global information. We compared the number of parameters and the speed of the above methods, as shown in Table 5.

**Figure 10:**
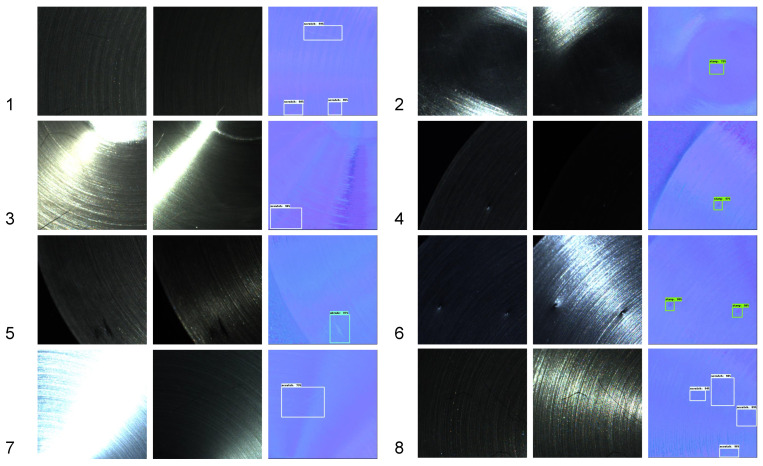
Diagram of detection results. We show the results of 8 sample. The numbers in the images represent the serial numbers of the samples. For each sample, we display 2 input images with the detection result.

It can be found that the Faster R-CNN had advantages in terms of speed and detection accuracy. Figure 10 shows a diagram of the detection results for different defects. We can see that our method was effective in detecting defects in the captured images that were disturbed by shadows or highlights. However, due to the information consumption in the photometric stereo, some defect areas were incompletely marked, such as sample 8 in Figure 10.

### 3.3. Detection Time Analysis

We took a stainless steel part with a size of 600×450 mm as the test object. Due to the large size of the part, it needed to be inspected in pieces. The PSDCE was driven by a manipulator and the detection took place in eight different workstations.

The detection times are shown in Table 6.

All processes were carried out in tandem, and it took about half a minute to complete the detection of a part. According to the result, our method is suitable for spot testing or offline testing. In future work, we will improve the method by parallelising the processes and shortening the prediction speed of the model to meet the real-time requirements of an industrial site.

## 4. Discussion

**Figure 11:**
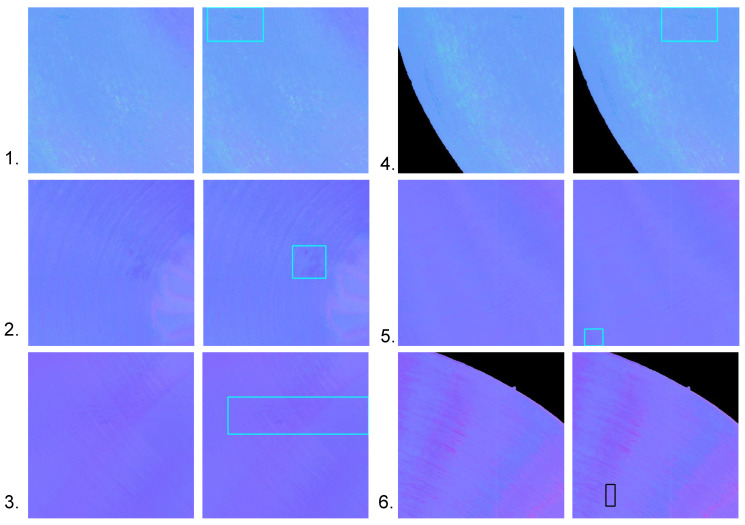
Detection failure cases. A total of 6 groups, of which groups 1–5 are abraded samples and group 6 is a scratch sample. The left image of each group is the estimated result and the right one is the ground truth. Boxes indicate the location of defects. For each group, the defect detection model did not identify the defect successfully.

As demonstrated by the experimentation shown previously, our system and dataset are effective for defect detection on a glossy surface. This is because the photometric stereo removes the effects of highlights and shadows by fusing the effective information from multiple directions of light and retaining only the normal information that is effective for defect detection. However, there are still some aspects which need improvement. We captured a small variety of objects in our dataset. In future work, we will capture metal parts in a variety of shapes and materials to expand the dataset and prevent overfitting of the model. We combined an existing photometric stereo model directly with the defect detection model during testing. Photometric stereo is not only able to obtain the normal map of the object but also to solve for the surface albedo. The defects mentioned above all vary in the depth direction and the albedo contributes less to defect detection, so we input the normal map directly into the detection model. However, when the photometric stereo model removes highlights, some texture information is inevitably lost at the same time, leading to a failure of the defect detection, as shown in Figure 11.

We can see that the smoothing operation of the convolution made some of the defects too indistinguishable from the background. In future work, we will improve the existing photometric stereo model to retain sufficient detail information when removing highlights and provide a priori information on the background to the defect detection methods. Meanwhile photometric stereo is also capable of obtaining an albedo map. Furthermore, for defects with insignificant changes in depth, the albedo is still valid information. Therefore, the integration of albedo information into the defect detection models is also an avenue for future research.

## 5. Conclusions

In this paper, we designed a photometric-stereo-based defect detection system (PSBDDS) to locate and classify the defects on metal parts and eliminate gloss interference. We used the PSBDDS to make a photometric defect detection (PSDD) dataset, which contained images under different illumination and corresponding light information. Based on the PSDD dataset, we proposed a framework for photometric-stereo-based defect detection, which could combine the existing state-of-the-art photometric stereo methods with a defect detection method. Experimental results showed that our system could effectively fuse valid information under multidirectional illumination, remove highlights and shadow interference, and improve the efficiency of defect detection on high-gloss surface objects such as metal parts.

## Figures and Tables

**Table 1:** The number of samples for each type of defect in the dataset.

	Stamp	Scratch	Abrade	Total
Training set	548	325	329	1202
Validation set	190	112	110	412
Test set	186	105	112	403

**Table 2:** Binary classification confusion matrix.

	Estimated	Positive	Negative
Ground Truth			
Positive		True positive (TP)	False negative (FN)
Negative		False positive (FP)	True negative (TN)

**Table 3:** Fast R-CNN performance comparison of different backbones on our PSDD datasets.

Backbone	mAP/%
VGG-16	16.4
ResNet-50	50.1
ResNet-50+FPN	75.5

**Table 4:** Performance comparison of different learning-based defect detection methods on our PSDD dataset. Numbers represent mAP (%).

Model	Stamp	Abraded	Scratch	Avg.
YOLOv3	35.5	13.6	33.3	29.5
SSD	54.9	45.1	48.9	50.9
Faster R-CNN	74.0	75.1	79.0	75.5

**Table 5:** Comparison of parameters and detection speed for different learning-based defect detection methods. The size of the test images was 400×400.

Model	Parameters	FPS
YOLOv3	62.6 MB	14
SSD	13.5 MB	4
Faster R-CNN	41.4 MB	11

**Table 6:** Time for each step of the system. Each step runs in tandem.

Manipulator Movement	PSDCE Work	Model Prediction	Total
10.5 s	8.8 s	24.8 s	36.1 s

## Data Availability

Not applicable.
